# Outpatient Ketamine Infusions for the Treatment of Fibromyalgia and Chronic Pain Syndrome: A Case Report

**DOI:** 10.7759/cureus.44909

**Published:** 2023-09-08

**Authors:** Sonakshi Sharma, Ruchir Gupta

**Affiliations:** 1 Pain Management, Mountain View Headache and Spine Institute, Phoenix, USA

**Keywords:** pain relief, case report, fibromyalgia, chronic pain, ketamine

## Abstract

Intravenous (IV) ketamine has demonstrable efficacy for chronic pain management. Its use in an outpatient setting has provided relief from myriad pain syndromes and additionally may reduce dependence on opioids. Nevertheless, its long-term ability to provide these benefits is understudied. Here, we present the case of a 68-year-old female who presented to our clinic with persistent fibromyalgia, accompanied by other pain symptoms that had been recalcitrant to traditional pain management therapy including nerve blocks, ablations, spinal cord stimulation, and morphine medication. We administered seven increasing IV ketamine doses over two weeks. The patient reported significant, widespread pain relief. The patient continued to receive IV ketamine twice weekly for over a year and remains on this schedule. Pain relief has persisted under this regimen, along with a demonstrable improvement in quality of life, a reduced use of morphine, and the cessation of anti-depressant medication. This case indicates that long-term ketamine infusions show promise for chronic pain management and that more longitudinal studies on this treatment are warranted.

## Introduction

Ketamine is an anesthetic agent that has been approved for clinical anesthesiology for over 50 years [1]. First synthesized in the 1960s, ketamine is a phencyclidine derivative that has the pharmacological effect of inducing general anesthesia while suppressing the reflexes of protective airways. In sub-dissociative doses, ketamine infusions, administered over the course of several minutes to several hours, have been found to produce analgesic effects that provide relief to patients with chronic pain [2]. While specific indications and protocols for ketamine infusion therapy are still under debate, infusions have been shown to provide pain relief benefits for fibromyalgia [3], central sensitization hyperalgesia [4], phantom limb pain [5], chronic migraines [6], and several other pain-related disorders [7]. Currently, there are research efforts examining ketamine treatment for chronic pain and refractory pain related to cancer, aiming toward more widespread usage of the therapy [7].

Despite the observations that ketamine is efficacious in modulating both localized and distributed pain, there is a lack of consensus on dosing regimens. The literature reports broad ranges of dosages per session and numbers of sessions, with no general agreement on optimization even among major medical associations [8]. Additional factors that contribute to uncertainty in dosing are that ketamine can be delivered through intravenous (IV) infusions, liquid solutions taken orally, or nasal spray; that ketamine has been delivered in a single dose or over a course of infusions lasting days, months, or even years; that ketamine is often given in combination with other analgesics; and that there exist both racemic and chiral pure formulations [1-4,7].

Here, we present the case of a 68-year-old female with fibromyalgia and central sensitization who had failed multiple conventional pain therapies such as spinal cord stimulation, sacroiliac (SI) joint fusion, numerous neuraxial injections, and nonopioid and opioid medications. The administration of IV ketamine was given over more than a year with a particular iterative dosage regimen. We describe the effects of long-term IV ketamine infusions on pain management, concomitant opioid and anti-depressant usage, general health, and mood. The objective of this case study is to demonstrate the efficacy of longitudinal ketamine infusions for refractory pain management and consequently increase the awareness of such treatment.

## Case presentation

A 68-year-old female presented to an outpatient pain management clinic for the evaluation of low back pain with radiation to bilateral lower extremities. The patient’s medical history was significant for fibromyalgia, chronic pain syndrome, central sensitization disease, degenerative joint disease, osteoarthritis, and bipolar disorder. The patient was diagnosed with fibromyalgia through a physician-conducted physical examination that indicated refractory generalized pain. She had been experiencing this pain for the past 10 years, during which she had attempted neuraxial injections, SI joint fusion, physical therapy, opioid and nonopioid pain medications, and spinal cord stimulator placement, all with little or no relief. The patient has no history of noncompliance with her medications. At the time of the initial consultation, the patient was already on a total of 105 mg morphine q24H (30 mg q12H and 15 mg three times a day {tid} as needed {PRN}) and regular chiropractic therapy exclusively for neck-related pain. The patient was evaluated as a 4 on the General Anxiety Disorder-7 (GAD-7) for anxiety and a 10 on the Patient Health Questionnaire-9 (PHQ-9) for depression. She undergoes a psychological evaluation every 30 days. The patient continued her chiropractic therapy during her ketamine infusions and stated that it provided relief for her pain, which is largely localized in the neck and lower back. High doses of opioid medication did not provide significant relief for the patient’s pain. She also described inadequate sleep and a weak appetite.

She described her pain as a constant stabbing and shooting that was an 8 out of 10 on the numerical rating scale (NRS, ranges from 0 to 10, with 10 being the most intense pain). Upon physical examination, the patient was in moderate distress. She had a positive bilateral straight leg raise test; flexion, abduction, and external rotation (FABER)/flexion, adduction, and internal rotation (FADIR) test; lumbar facet loading test; bilateral Yeoman’s test; and bilateral Stinchfield’s test, indicating pain in the sacroiliac and hip regions. The patient also had diffuse pain located in her neck.

**Figure 1:**
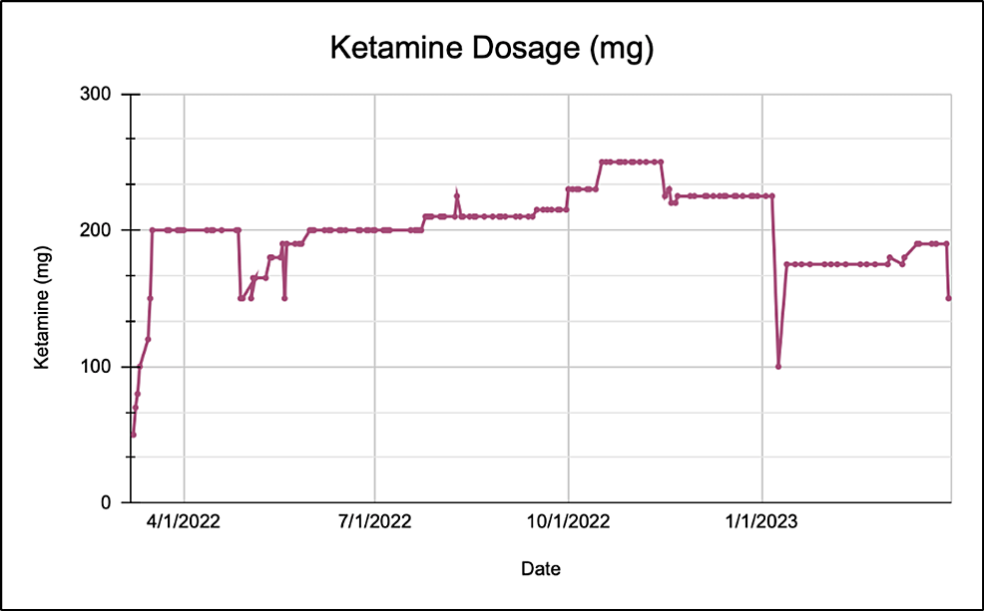
The patient’s ketamine dosage in milligrams over the course of one year of infusions.

The patient initially received a series of seven IV ketamine infusions, one hour in duration, once a day over the course of two weeks. The patient was started on a 1.0 mg/mL ketamine concentration, 0.5 mg/kg/hour infusion rate, for a total of 50 mg of delivered ketamine. She did not exhibit signs of psychosis or altered mental status during or after the infusion. Her dosages were adjusted frequently based on her tolerance of IV ketamine and whether any analgesia was noticed. The patient’s dose for her second infusion one day later was raised to 1.4 mg/mL ketamine concentration, 0.7 mg/kg/hour infusion rate, for a total of 70 mg of ketamine dosage. For days 3-7, the patient’s dosages were as follows in milligrams: 80, 100, 120, 150, and 200, all administered over one hour each (Figure 1; Table 1). After the first seven infusions, the patient stated that she felt a significant amount of pain relief, noting that she was surprised that all her areas of pain were impacted by the ketamine.

**Table 1:** The first seven infusions along with the ketamine concentration, infusion rate, and total dose.

Infusion Date	Ketamine Concentration (mg/mL)	Ketamine Infusion Rate (mg/kg/hour)	Total Ketamine (mg)
03/08/2022	1	0.5	50
03/09/2022	1.4	0.7	70
03/10/2022	1.6	1	80
03/11/2022	2	1.2	100
03/15/2022	2.4	1.3	120
03/16/2022	3	1.3	150
03/17/2022	4	1.6	200

At a one-month follow-up, the patient stated that she started experiencing >50% pain relief that contributed to a significant improvement in her quality of life. Her activities of daily living also improved with an increased ability to cook meals and socialize with family and friends and improved sleep (the latter for the first time since the onset of her pain 11 years prior).

Monthly maintenance ketamine infusion therapy was administered to the patient with her dose and rate adjusted to the level of maximal improvement (Table 2), with the highest dosage being 250 mg.

**Table 2:** The number of infusions, the range of infusion dosages, and the combined monthly ketamine infusion dosage over the course of one year.

Month	Number of Infusions	Range of Ketamine Dosage (mg)	Total Ketamine Dose (mg)
March	12	50-200	1770
April	9	150-200	1700
May	14	150-200	2485
June	13	200	2600
July	14	200-210	2840
August	14	210-225	2955
September	12	210-215	2555
October	14	230-250	3360
November	12	220-250	2820
December	12	225	2700
January	8	100-225	1425
February	6	175	1050
March	10	150-190	1810

At the one-year mark, the patient continues to be maintained at eight infusions per month, twice weekly. She is at a 3.5 mg/mL infusion concentration, a 1.1 mg/kg/hour infusion rate, and a 175 mg dosage per infusion. Kidney function laboratory results show a 16 mg/dL blood urea nitrogen (BUN) and a 0.9 mg/dL creatinine. The patient’s aspartate transaminase (AST), alanine transaminase (ALT), alkaline phosphatase (ALP), albumin, and bilirubin are within range currently. Serial urine drug screening (UDS) results have also been consistent with patient-reported medications.

Furthermore, the patient’s morphine dose has decreased by 43%, from 105 mg a day to 60 mg a day. Her Patients’ Global Impression of Change (PGIC) score since the start of infusions is 1, and she continues to describe a 50% improvement in pain since the beginning of the infusions. Previously taking Lexapro to treat her bipolar disorder, the patient ceased this drug, noting that the ketamine infusions have also helped her manage the disorder (Table 3).

**Table 3:** The patient’s medication dosage before and after starting ketamine infusions. PGIC: Patients’ Global Impression of Change

Medication	Dosage Pre-infusions	Dosage Post-infusions
Morphine	105 mg	60 mg
Lexapro	100 mg	0 mg
PGIC	7	1

## Discussion

Though its pathophysiology has not been comprehensively elucidated, ketamine is known to be a noncompetitive antagonist to N-methyl-D-aspartate (NMDA) receptors, which are ligand-gated channels for glutamate, an excitatory neurotransmitter [9]. It has been found that the firing of nociceptors releases glutamate, activating spinal cord NMDA receptors and resulting in central sensitization [10]. Studies conducted on nonhuman pyramidal cells and spinal cord neurons have shown that ketamine is capable of blocking excitatory postsynaptic action potentials [11,12]. The muscle pain and deep hyperalgesia characteristic of fibromyalgia are rooted in central hyperexcitability, which can be modulated by NMDA receptors and, consequently, NMDA receptor antagonists such as ketamine [1]. Following these findings, investigating the usage of ketamine as a pain management method for chronic pain seems useful [13].

Ketamine has several routes of administration, with intravenous administration producing a bioavailability of 100% [9]. Current protocols suggest a series of 1-5 inpatient infusions for patients with chronic pain [13]. The present case demonstrates successfully that ketamine can be used for refractory pain over a long period of time. Long-term ketamine therapy has presented concerns such as hepatotoxicity [14]. The current patient’s within-range BUN, creatinine, and liver function levels, however, indicate normal functioning. We believe that maintaining the patient’s vital signs in the normotensive zone is essential to preventing any end-organ damage.

In the current case, ketamine infusions have also helped the patient reduce morphine and Lexapro use (Table 3). The cumulative monthly ketamine dosage administered to our patient did not progressively increase over the year-long treatment, undermining the notion that subsequent ketamine treatments require progressively higher dosage as a consequence of the development of tolerance (as seen in opioid use). Rather, the cumulative monthly ketamine dose increased to a maximum of 3360 mg in October and decreased to 1810 mg the following March, despite consistently providing >50% pain relief (Table 2).

The tolerance that opioid medications generate in patients being treated for chronic pain has contributed to the declaration of the opioid epidemic as a public health emergency within the United States [15,16]. Thus, while the patient received 18675 mg of ketamine during the overall course of her therapy, this amount must be counterbalanced by the 23520 mg of morphine she would have taken had her morphine dose not been reduced because of the ketamine infusions. We believe that compared to the adverse side effects and addictive potential of opioid medication, ketamine is a safer therapeutic option. Additionally, IV ketamine is administered in a monitored setting where vital signs can be maintained within a normal range. Orally taken morphine has no such surveillance associated with its use. It is important to note that ketamine or opioid medications were not the only therapeutic avenues pursued by the patient; alongside her regular ketamine infusions, the patient continued her chiropractic therapy and mental health care. In addition, she noted that SI joint fusion, neuraxial injections, and spinal cord stimulation did not provide benefit toward her pain. The patient is a homemaker and does not have an occupation that could aggravate her pain.

In addition to reduced opioid usage, the patient also stopped her Lexapro medication, which she had been taking for bipolar disorder. Research shows that ketamine infusions have benefited those diagnosed with treatment-resistant depression and bipolar disorder [17,18]. Thus, the relationship between the patient’s ketamine infusions, chronic pain, and bipolar disorder is difficult to definitively delineate. Further research examining the contributions of the ketamine therapy toward psychiatric versus musculoskeletal disorders would be useful. The patient’s ketamine infusions helped both her chronic pain and her bipolar disorder symptoms indicating that ketamine has multiple functionalities and can be used to treat more than one condition at a time. It is difficult to quantify the benefit that the patient’s improved quality of life has had on her health; however, positive mood and social effect in patients are related to improvement in overall patient health outcomes [19,20].

## Conclusions

The usage of longitudinal ketamine infusions for refractory chronic pain has shown significant and lasting benefits for this patient. This therapy has improved not only the patient’s chronic pain but also her mental health and medication dosage. Future clinical studies investigating the long-term impact of ketamine infusions on different aspects of a patient’s life would be incredibly beneficial. As the opioid epidemic continues to impact communities globally, research into nonopioid alternatives for chronic pain management is an important endeavor.
